# Pandemic influenza viruses: time to recognize our inability to predict the unpredictable and stop dangerous gain-of-function experiments

**DOI:** 10.1002/emmm.201303475

**Published:** 2013-10-24

**Authors:** Simon Wain-Hobson

**Affiliations:** 1 Institut PasteurParis, France; 2 The Foundation for Vaccine ResearchWashington, DC, USA

**Keywords:** gain-of-function, influenza, pandemic, vaccines, virus

»There are known knowns. There are things we know we know. We also know there are known unknowns. That is to say we know there are some things we do not know. But there are also unknown unknowns, the ones we don't know we don't know.«

D.H. Rumsfeld, U.S. Secretary for Defense, February 12, 2002

For almost as long as the civil war in Syria, the scientific community has been locked in a controversy about the utility of gain-of-function (GOF) research to increase the transmissibility of highly pathogenic avian influenza H5N1 viruses that resulted in a year-long moratorium (Nature, 2012a, b, 2013a, b). Now, for the first time since the controversy erupted in November 2011, there is a growing recognition among scientists that GOF virology – experiments with H5N1 and now H7N9 and H7N7 viruses – will **not** help us predict a pandemic (Morse et al, 2012; Merson et al, 2013), nor will it help us develop more effective vaccines (Butler, 2012; Malakoff, 2013), the two principal arguments for doing the research. Chinese officials have gone one step further by signalling their view that GOF research has no benefit for humanity (Malakoff, 2013; http://www.handelsblatt.com/technologie/das-technologie-update/healthcare/vogelgrippe-manipulierte-viren-sind-gefahr-fuer-menschheit/8611922.html).

There will be no end to the GOF saga until virologists, epidemiologists and public health officials acknowledge our fundamental inability for the moment to predict the unpredictable and until policy makers and funders move to rein in their support for GOF research.

## Known knowns

To start its business, a virus generally has to replicate well. This process isn't invariably accompanied by pathology, but it often is. For a virus the key factor is transmission because without it the virus goes to extinction very quickly. Transmission studies, which have been the poor cousin in virology, incorporate features such as droplet size, coughing, sneezing and virus load (Sorrell et al, 2011), which are not readily amenable to analysis using a mutagenesis kit. With an animal model, the first question is – or should be – how good is the model, especially given the old adage that the best model for man is man? The other handicap is time. Pathology and transmission studies take time, include large numbers of variables, while correlations are easier to establish than proof. Molecular analyses of virus replication or virus–host interactions in a tumour cell line are simpler and faster. While fair game 20 years ago, there is more to a viral disease than just the virus. There is evolution and the big conundrum as to why one virus, or one strain, goes pandemic, while another doesn't.

In the modern gene era, say post 1980, we have only known two new pandemics, AIDS and H1N1 influenza. Yes, human hepatitis B and C continue to exact huge tolls, as does measles, but virology came to them as established global diseases. HIV/AIDS was pre-PCR, pre-genomics and pre-high-throughput, although the scientific advances made between 1983 and 1988 were without precedent. It transpires that HIV-1 crossed over to humans in the early part of the 20th century (Salemi et al, 2001), and so the subtleties of just how a chimpanzee virus morphed into a human virus producing a pandemic are lost on us. Virologists have shown themselves to be very creative in exploring why HIV-1 subtype M went global, as opposed to HIV-1 subtypes N, O, P and HIV-2, all of which succeeded to various degrees yet didn't pan out (Kirchhoff, 2009). We can surmise, perform some clever viral archaeology (Worobey et al, 2008), but little more.

Which brings us to influenza. The spring of 2009 saw a series of cases of H1N1 human influenza in Mexico. The specter of a pandemic caused by the so-called swine flu shocked scientists and health officials worldwide and a massive investment was made in purchasing antivirals and developing a vaccine in record time. Fortunately it turned out to be a mild pandemic. Up to then, the general feeling was that the next pandemic virus would emerge from Southeast Asia, so a Central American epicentre foxed everyone. As to timing, if you repeat often enough that a pandemic is around the corner, something I have heard regularly since getting into human virology back in 1980, then one day you'll be right. Human flu pandemics occurred in 1918, 1957, 1968 and 2009, so number theory alone suggests that the next one is due anytime between 2020 and 2050. This is not to make fun of the virologists who have treated us to a fabulous decade of influenza science; what it does is neatly capture the difficulties in predicting viral emergence of human-adapted strains capable of causing a pandemic (Holmes, 2013).

Influenza A viruses infect birds and mammals. Their segmented genomes allow for extensive gene swapping, or reassortment in flu jargon. Birds harbour by far and away the largest reservoir of different avian influenza viruses encoding most of the 17 known haemagglutinins (H) and the 9 neuraminidases (N), which are the surface proteins that allow the virus to bind to, and break away from the cell. Because these proteins are so important and readily distinguished serologically, viruses are referred to as H1N1, H7N9 and so on. In the past humans have been plagued by H1N1, H2N2 and H3N2 viruses.

Other avian influenza viruses also cross over to humans. They can come from poultry, such as H5N1 or H7N7; or where the original source may be birds or waterfowl, *e.g*. H7N9, the virus can cycle through live poultry markets, as we saw earlier this year in China (Wang et al, 2013). All these viruses *can* be highly pathogenic in humans. The case fatality rate for individuals with symptomatic infection can be as high as 60%. Fortunately these infections rarely result in transmission from humans to other humans, although short transmission chains involving a handful of individuals have been reported (Zaman et al, 2011; Qi et al, 2013). This apparent lack of human-to-human transmission among avian influenza viruses is strongly influenced by the haemagglutinin, which cannot bind the α-2,6 linked sialylated glycan receptors in the mammalian upper respiratory tract, while pathology results from the infection of cells bearing α-2,3 linked sialylated glycans in the lower tract. This follows from the fact that avian haemagglutinins naturally use the α-2,3 linked sialylated glycan as receptors. Accordingly, adaptation to mammals requires mutations allowing the virus to bind the mammalian sugar. Other mutations may be necessary to permit avian influenza viruses to grow efficiently, but for simplicity we will focus on the haemagglutinin.

…all the evidence indicates that proponents of GOF research have greatly overstated the benefits while the risks remain.

Influenza A viruses are mutation machines. Like all RNA viruses – poliovirus or hepatitis C virus – their RNA genomes are replicated without any proofreading mechanisms. Their intrinsic mutation rates are approximately one mutation per round of replication, which is close to the maximum possible. Hence the possibility is real that over time, one day, a novel avian influenza A virus could mutate into a robust human-transmissible virus sparking a pandemic. As the antigenic surface of a novel virus such as H7N9 would be totally new, there would be no pre-existing immunity in the human population to blunt virus replication. Such cases are referred to as *antigenic shift*, as opposed to *antigenic drift* where the annual virus mutates, typically in one antigenic site. Antigenic shift is correlated with a human pandemic.

## Known unknowns

In some ways it is akin to an excruciating waiting game. Where and when and from which species will the next flu virus jump to humans and how severe will the pandemic be? Waiting for and focusing on the big one overlooks the fact that viruses are constantly challenging us. Indeed, every living species on the planet is under threat from several viruses. For example, there are a host of viral infections that are not passed to other individuals. The virus dies out in what is called a dead-end infection. The most well known is rabies where there are 55,000 human deaths per year worldwide. By comparison lymphocytic choriomeningitis, hendra, nipah and hantaviruses are small fry. Fortunately for humans, these viruses have other hosts.

This brings us back to transmission. Dead-end infections and limited infections are part and parcel of viruses' unstoppable propensity to infect, whether the infection is abortive or not. Yet these RNA viruses are mutation machines with the potential to adapt to a new host. How many of them will morph, say, in the next 100 years? We simply don't know. The recent H7N9 influenza outbreak – essentially all dead-end infections – took everyone by surprise. Nobody predicted the SARS or MERS-coronavirus outbreaks; the former was clearly accompanied by human-to-human transmission, while the latter seems to result mainly in dead-end infections.

Microbes will always be testing new niches. What we have to try and work out is which viruses have the greatest probability to adapt to humans and which ones will remain minor players. Prediction is complicated by the fact that evolution is full of contingency, while some lineages become extinct. While each death is a terrible loss, the morbidity and mortality caused by H5N1, H7N9, MERS-coV, nipah and hendra viruses are mere tremors. By contrast pandemics are rare events. Few viruses make it big time, while lesser shocks will be more frequent. The parallel with earthquakes is obvious – the bigger the quake, the less frequent they are. By contrast, tremors are commonplace.

Against this backdrop a small group of influenza virologists conceived the hypothesis that the forced evolution or the deliberate engineering of highly pathogenic avian influenza A H5N1 viruses, which are not currently transmitted easily to and between humans, could help them predict the mutations necessary to permit efficient aerosol transmission between ferrets, the preferred model for influenza transmission studies. Pandemic human strains are transmissible between ferrets by the aerosol route. This work, later dubbed gain-of-function (GOF) research, was touted as being informative in terms of helping us predict the next pandemic strain, develop more effective vaccines, design better drugs, as well as improve pandemic preparedness and field surveillance.

Using different viral starting points, three groups in the USA, the Netherlands and China succeeded in generating viruses that were efficiently transmitted between ferrets. Adaption was accompanied by a number of mutations, some of which were common, others unique (Herfst et al, 2012; Imai et al, 2012; Zhang et al, 2013). When the studies became known on the conference circuit they caused an uproar. A flood of questions emerged ranging from biosafety concerns, to whether the mutations should be published, which would make the information accessible to all, bioterrorists included (Hanley, 2013), while other scientists questioned the significance and robustness of the results in terms of virus evolution.

Rather than go into a fascinating historical narrative, let's stick to the virology and the claims made by proponents of GOF research.

A selection screen will give you what you are looking for, provided the initial mutant population is large enough. If you continuously select virus from aerosol-infected ferrets with respiratory distress you will end up with a highly transmissible and pathogenic virus. If animals with asymptomatic infections are chosen, the resulting virus will be a highly transmissible virus with low pathogenic potential (Wain-Hobson, 2013). The question that nobody can answer is exactly what genetic configuration nature will come up with, say, if the H5N1 virus – or the H7N9 virus – were ever to solve the problem of efficient human-to-human transmission by themselves? The power of experimental selective screens must not be underestimated. A spectacular molecular example is the recovery of ribozymes from pools of random RNA molecules (Bartel & Szostak, 1993). A very different example is the domestication of the Siberian silver fox. As a result of selective breeding over 50 years the foxes became tamer (Trut, 1999).

Since the H5N1 GOF studies were forced evolution experiments we simply have no way of knowing if evolution will take any of the trajectories suggested by GOF research, or whether any of these viruses could set up a pandemic. The experiments can suggest combinations of mutations but they can't prove their case because the deliberate infection of humans is unthinkable. Even if the viruses did infect humans via the aerosol route and replicated well, would they set up a pandemic or merely lead to regional outbreaks, like the SARS and MERS coronaviruses? In Popperian terms the influenza GOF experiments are unfalsifiable.

If GOF virology cannot deliver any benefits, while the catastrophic risks are tangible and remain, common sense suggests that it should stop.

In terms of vaccination, adaptation of an avian influenza virus to human cells might – and probably would – impact antigenicity. Indeed, many vaccinologists consider that antigenicity would be impacted. For the moment, matching the vaccine strain as closely as possible to the circulating strain remains the tried and tested solution for developing flu vaccines. Several observers, quoting expert sources, have reported that vaccine makers consider there is little in this influenza GOF research that will help them develop more effective vaccines (Butler, 2012; Malakoff, 2013). This is important because proponents of GOF research have been very vocal in making this claim.

Concerning antiviral drugs, GOF viruses are not indispensible; circulating wild-type viruses are perfectly good enough to test available and experimental drugs. We can only hope that the next pandemic influenza virus will be sensitive to one or more of these drugs. From the perspective of an HIV virologist who witnessed the failure of monotherapy and the triumph of tritherapy, the use of a single drug against a mutation machine, whether HIV or influenza, will inevitably result in widespread drug resistance, it being simply a matter of time.

What about pandemic preparedness? Modern virology, technology and the interconnection with public health is pretty good. The Chinese epidemiologists, virologists and public health authorities did a remarkable and very professional job picking up very fast the recent H7N9 infections and stamping out transmission, even though the reservoir is still unidentified. Similarly with just a handful of cases, the MERS-coronavirus was identified very quickly (Zaki et al, 2012). There is no doubt that with more experience virology, epidemiology and public health will dovetail even more.

## Unknown unknowns

As to surveillance, there are too many diverse viruses out there to have a fail-safe surveillance system, even if enormous resources were poured into strengthening such networks. There are more viruses on the planet than cells (aka bacteria). Their genomes range from 2 to 240 kb for mammalian viruses and up to 2.5 Mb for viruses that prey on unicellular eukaryotes, all of them showing stunning genome variation. Metagenomic studies are identifying bewildering numbers of previously unidentified viruses on human skin and elsewhere. If non-human mammalian, avian and insect-borne viruses alone are factored in as springboards to humans, the number that could possibly cross over is essentially limitless. Virology is replete with case reports of unusual viruses, one of the most remarkable examples being a recombinant of a papilloma and polyoma virus, which is decimating an endangered species of Australian bandicoot (Woolford et al, 2007).

The RNA viruses are formidable because of their very high mutation rates; and, in the case of influenza viruses, their ability to reassort, producing phenomenal numbers of distinct viruses. The recent H7N9 influenza virus is believed to be a reassortant of four bird, waterfowl or chicken viruses, while a recent H5N2 influenza virus from Vietnam was derived from three parents (Li et al, 2013; Nishi et al, 2013). Paying particular attention to RNA viruses with the potential to cause human respiratory disease makes good sense because more than half of humanity is now urban, although the history of the spread of HIV/AIDS mustn't be forgotten. Testing fixed mutations spotted in field isolates of influenza viruses – those found at reasonably high frequencies that distinguish them from the general mutational background – isn't a problem. These mutations can be rapidly introduced into a reference strain or the local strain if the genetic background is thought to be an issue and aerosol transmission tested. Such studies are real world and have the merit of producing tangible information that public health officials can assimilate.

Taken together, all the evidence indicates that proponents of GOF research have greatly overstated the benefits while the risks remain. Scientists are generally upbeat about their work yet notoriously underestimate risk (Van Noorden, 2013). Safety experts know full well that no system is perfect, a truism that takes us back to high school physics classes. So what are the risks? Escape from a high-containment facility or infection of a lab worker leading to community spread are among the more prosaic (Lipsitch & Bloom, 2012). And then there's insider risk – as witnessed in the anthrax attacks – which no one wants to talk about (Culp, 2013). While the probability of an accidental or deliberate release of a human-transmissible virus from a single lab is arguably small, but not zero (Henkel et al, 2012), the more groups performing GOF virology, the greater the overall risk. In the nuclear area all efforts are concentrated on reducing the proliferation of labs processing and handling fissile material.

The big unknown of course is whether an escaped GOF influenza virus, or any other virus for that matter, would set off a pandemic, for as we have seen predicting a pandemic strain is currently beyond our grasp. However, what we can do is compute the outcome of risk scenarios using sophisticated modelling systems that use a variety of plausible basic reproductive ratios (*R*_0_) and case fatality rates, *etc*. The projected numbers for mortality, morbidity and economic impact are not pretty (Verikios et al, 2011). This study is particularly pertinent as it shows that a virus of lower pathogenicity but higher transmissibility has greater impact on humanity than a highly pathogenic, but poorly transmissible virus. So any experiment that makes H5N1 or H7N9 more transmissible between ferrets, and presumably humans, even at the loss of some pathogenicity, is making the world a more dangerous place than it presently is with sporadic outbreaks of H5N1 and H7N9 influenza. In this respect it is staggering that, two years into the biggest controversy in virology in decades, no thorough risk analysis has been conducted. Simply staggering.

GOF influenza research may well be just one small step for virology; the problem is it's a giant risk for mankind.

Viral transmission, adaptation to new hosts and emergence are crucial issues in virology and we need to know far more. Yet given the present corpus of knowledge and all the unknowns, there is nothing in GOF virology that will help us predict a pandemic or help us develop more effective vaccines. It is tantamount to reckless playing with fire.

If GOF virology cannot deliver any benefits, while the catastrophic risks are tangible and remain, common sense suggests that it should stop. GOF influenza research may well be just one small step for virology; the problem is it's a giant risk for mankind.
